# Individualised behaviour change strategies for physical activity in multiple sclerosis (IPAC-MS): protocol for a randomised controlled trial

**DOI:** 10.1186/s13063-019-3768-7

**Published:** 2019-12-02

**Authors:** Farren L. Goulding, Charity D. Evans, Katherine B. Knox, Hyun J. Lim, Michael C. Levin, Sarah J. Donkers

**Affiliations:** 1 College of Pharmacy and Nutrition, University of Saskatchewan, 104 Clinic Place, Saskatoon, SK Canada; 2 College of Medicine, University of Saskatchewan, 107 Wiggins Road, Saskatoon, SK Canada

**Keywords:** Multiple sclerosis, Exercise, Physical activity, Individualised, Behaviour change strategy, Neurophysiotherapist, Randomised controlled trial

## Abstract

**Background:**

Multiple sclerosis (MS) is a chronic, degenerative disease of the central nervous system. Because of the long-term and unpredictable nature of the disease, the burden of MS is significant from both a patient and societal perspective. Despite a recent influx of disease-modifying therapies to treat MS, many individuals continue to experience disability that negatively affects productivity and quality of life. Previous research indicates that physical activity has a positive impact on walking function in individuals with MS, in addition to the usual beneficial effects on overall health. However, most people with MS are not active enough to gain these benefits, and a lack of support to initiate and maintain physical activity has been identified as a major barrier. This study will evaluate the impact of a novel intervention involving individualised behaviour change strategies delivered by neurophysiotherapists on increasing physical activity levels in individuals with MS who are currently inactive.

**Methods/design:**

This single-blind, parallel-group, randomised controlled trial will be conducted in Saskatchewan, Canada. Eligible participants include individuals with MS who are ambulatory but identified as currently inactive by the self-reported Godin Leisure-Time Exercise Questionnaire (GLTEQ). The intervention will be delivered by neurophysiotherapists and includes individualised behaviour change strategies aimed at increasing physical activity over a 12-month period. The control group will receive usual care during the 12-month study period. The primary outcome is the change in physical activity level, as measured by the change in the GLTEQ score from baseline to 12 months. Secondary outcomes include the change in patient-reported outcome measures assessing MS-specific symptoms, confidence and quality of life.

**Discussion:**

Physical activity has been identified as a top research priority by the MS community. Findings from this novel study may result in new knowledge that could significantly impact the management and overall health of individuals with MS.

**Trial registration:**

ClinicalTrials.gov, NCT04027114. Registered on 10 July 2019.

## Introduction

Multiple sclerosis (MS) is a chronic degenerative neurological disease involving the central nervous system. Symptoms of MS are unpredictable and can affect multiple body systems. The disease may be broadly categorised as relapsing-remitting or progressive [1]. Over time, most cases follow a progressive course [2], and an estimated 50% of individuals with MS require a cane within 15 years of disease onset [3]. There is no cure for MS, and although several different disease-modifying therapies are available, there is still controversy about their long-term effectiveness, and they are not indicated for all individuals with MS [4].

In the general population, people who are moderately physically active have a lower risk for medical co-morbidities and an increase in lifespan by an average of 7 years [5]. Physical activity has been shown to have considerable benefit in MS, regardless of disease type or duration [6]. Physical activity has also been proposed to have a disease-modifying impact [7], supported by magnetic resonance imaging outcomes [8] and research in animal models [9]. Regardless of evidence supporting a positive effect, less than 20% of individuals with MS are sufficiently active for health benefits [10]. Despite the known benefits of exercise in MS, there has been little change reported in physical activity levels in the MS population over the past 25 years [11]. Historically, the standard approach for promoting physical activity in MS research has involved structured exercise training [12]. However, individuals with MS routinely cite a lack of support and resources as a major barrier to regular physical activity [5]. A recent review on exercise interventions in MS identified the largest effect sizes for increasing physical activity were from those involving behaviour change strategies [13]. The same review noted that existing behaviour change strategy research was difficult to replicate and implement in clinical practice due to insufficient detail about the actual interventions [13].

In order to better describe the active components of behaviour change interventions and adopt the most effective behaviour change strategies, the Behavior Change Technique Taxonomy (BCTT) was created [14]. The BCTT includes a comprehensive list and definitions of behaviour change strategies. Behavior Change Theory describes the ‘why’ and informs selection of behaviour change strategies, or the ‘how’ [15, 16]. Few studies have applied theories of behaviour change in MS physical activity interventions [17], and those that have were focused largely on ‘packaged’ rather than individualised behaviour change interventions [18]. For example, fatigue in MS is identified as a major barrier to exercise [19]. A study may deliver a behaviour change intervention through a well-designed fatigue self-management programme; however, not every individual with MS will have fatigue as their main barrier to physical activity. Therefore, individualised behaviour change strategies that address each person’s unique and most significant barriers are recommended; yet, few have been applied in MS physical activity research to date [20].

Although effective at increasing physical activity behaviour, behaviour change strategies alone are believed to account for only 20% of change [16]. A recent study on behaviour change interventions recommended the added value of professional support [21], because individuals with MS benefit from the intermittent support of a specialist with expertise in exercise and MS to help maintain activity levels and function as the disease progresses [22]. The purpose of this study is to evaluate if a novel intervention of individualised behaviour change strategies delivered by neurophysiotherapists with expertise in MS increases physical activity levels in individuals with MS who are currently inactive.

## Methods/design

### Study design and setting

The IPAC-MS (Individualized Physiotherapy and Activity Coaching for Multiple Sclerosis) study is a prospective, single-blind, parallel-group, randomised controlled trial conducted in the Canadian province of Saskatchewan. The study is designed as a superiority trial and is a collaborative effort of interdisciplinary researchers, clinicians, and patient and family advisors Additional file 1.

### Study participants

Participants will be recruited primarily through the Saskatchewan MS Drugs Program (SMSDP). The SMSDP is a provincial initiative created to oversee the applications of all individuals applying for government coverage of a disease-modifying therapy for MS. At the time of enrolment in the SMSDP, individuals are offered the chance to consent to be contacted about participating in future MS-related research; those who consent are also asked to complete the Godin Leisure-Time Exercise Questionnaire (GLTEQ) [23–25]. Approximately 50% of all applicants to the SMSDP have consented and completed the GLTEQ. Individuals with a GLTEQ score < 24 are considered not sufficiently active for substantial health benefits and will be the primary cohort targeted for recruitment. If necessary, recruitment may also occur through the Saskatoon MS Clinic, the primary referral site for all Saskatchewan patients with MS, and through local MS Society organisations.

Individuals older than 18 years of age, with clinically definite MS, having a Patient Determined Disease Steps score ≤ 6 (i.e., able to walk with or without aids) [26], and who are estimated not to be sufficiently active for substantial health benefits (i.e., self-reported exercise less than four times weekly) are eligible for this study. Those who are unable to provide consent or are deemed to have a moderate-high risk for exercise-related harm based on the Physical Activity Readiness Questionnaire [27] will be excluded.

### Randomisation and blinding

All consenting participants who have completed a baseline assessment will be stratified into one of three categories based on their baseline GLTEQ score (< 9, 9–17, ≥ 18). Participants from each of the three strata will be randomly assigned to either the intervention or control group by a centralised telephone request to a study coordinator not involved in data collection or analyses. Randomisation lists were computer-generated in blocks of 4 to help achieve balance in the groups. The randomisation list was created prior to participant recruitment by a research team member not involved in data collection or analyses. Given the nature of the intervention, only the outcome assessors involved in the data collection will be blinded to the study group.

### Intervention and control

The intervention is an individualised physical activity behaviour change programme. The intervention will be delivered by neurophysiotherapists with expertise in MS over a 12-month period. Because each programme is specifically created for each individual, components of the intervention may vary between participants. However, there are three consistent features: behaviour change strategies, recommendations for physical activity, and ongoing neurophysiotherapist support. After randomisation, participants in the intervention group will undergo a tailored intake by a neurophysiotherapist, which will serve as the foundation for the individualised approach. At the initial intake, neurophysiotherapists will evaluate the participants’ individual attributes and physical activity needs in addition to a general physiotherapy assessment (e.g., MS symptoms and functional levels) to create personalised programmes. This initial intake may occur over one to three sessions and in variable formats, depending on the participants’ needs. To promote consistency, neurophysiotherapists will be trained in the Behaviour Change Wheel [28], the BCTT [14], and the Social Cognitive Theory of Behaviour Change, including correlates and determinants of physical activity behaviour in MS [20]. All training of the neurophysiotherapists was done with a standardised programme and delivered by the same instructor.

All physical activity recommendations made by the neurophysiotherapists will be based on established guidelines, existing resources, and individual participant needs. Best practice guidelines recommend that individuals with MS who have mild to moderate disability work up to at least 30 minutes of moderate intensity aerobic activity twice per week and resistance exercises for major muscle groups twice per week [29]. Support from a neurophysiotherapist will be available to the intervention group throughout the study period. We have allotted 15 neurophysiotherapist contact hours per participant, and each encounter will be recorded (method of contact, service delivered, and time required) using standardised data collection forms. The neurophysiotherapist support may occur in person, via telephone, using web-based methods and/or by telehealth. The neurophysiotherapist will record the types of behaviour change strategies used with each participant according to the framework and descriptors from the BCTT [14]. Intervention participants will also receive printed educational material from the MS Society of Canada on physical activity [29], diet [30] and stress management [31] at 2, 4 and 8 months. Because participants are involved in developing their own treatment plans, good adherence to the intervention is expected.

**Fig. 1:**
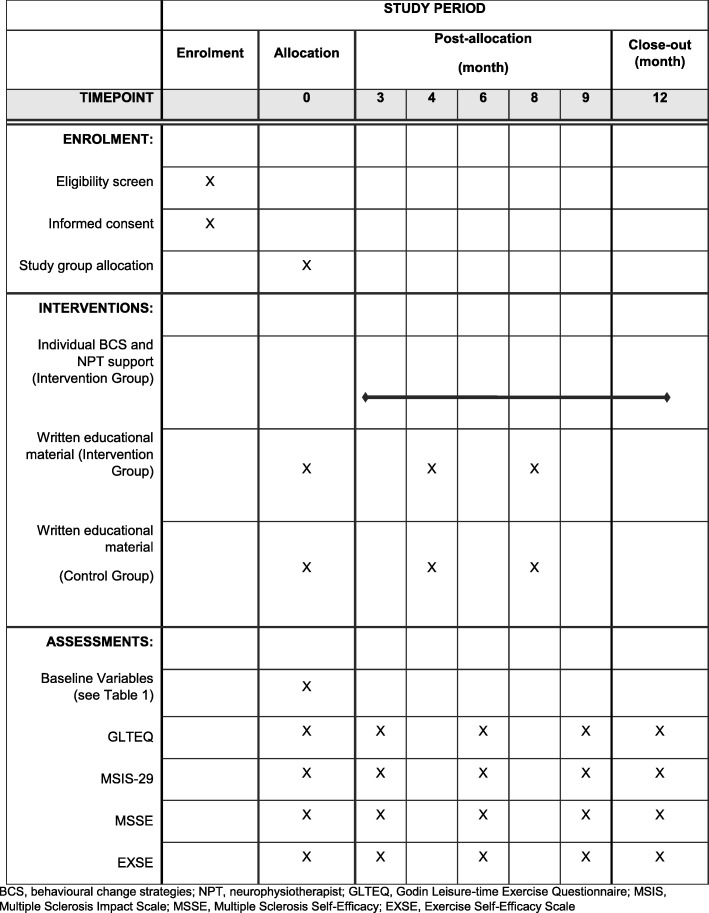
Schedule of study enrolment, interventions, and assessments

The control group will receive the same printed educational material as the intervention group at 2, 4 and 8 months. Participants in the control group will not receive any individualised assessments or recommendations, nor will they have access to neurophysiotherapist support throughout the 12-month study period (Fig. 1). At the end of the study, the control group participants will have the opportunity to receive the intervention; however, any results generated will not be part of the primary study analyses.

### Study outcomes

The primary outcome is the change in physical activity level, as measured by the change in the GLTEQ score from baseline to 12 months. The GLTEQ is a validated self-reported measure of physical activity with sensitivity to detect change in MS exercise interventions [23, 24]. The GLTEQ requests activity levels in the week prior (minimising recall bias), is easy to administer, demonstrates good test-retest reliability in ambulatory individuals with MS, and correlates with other more costly and/or less convenient measures of physical activity in MS, such as accelerometers [32]. The total GLTEQ score is calculated according to the number of self-reported strenuous, moderate- or mild-intensity physical activity in 15-minute time blocks over the week. A total leisure activity score is then computed, which includes a correction for the estimated metabolic demands associated with these intensity levels. The total GLTEQ score (range, 0–119) is recommended for use in physical activity research in MS [25], with higher scores indicating greater activity levels. A score > 23 is considered sufficiently active for substantial health benefits [23, 25].

Secondary outcomes include the change in patient-reported outcome measures assessing MS-specific symptoms, confidence and quality of life. The Multiple Sclerosis Impact Scale v2 [33] is a self-administered questionnaire evaluating patient-perceived physical and psychological impact of their MS. The Multiple Sclerosis Self-Efficacy Scale is an MS-specific, self-reported, self-efficacy measure with strong psychometric properties [34]. The 18-item version will be used whereby participants rate their level of confidence regarding components of disease management using a 10-point scale (very uncertain to very certain). Self-efficacy has been identified as one of the most consistent correlates of physical activity [35]. The Exercise Self-Efficacy Scale [36] is a validated and reliable measure for MS. It is a patient-reported ordinal six-item measure whereby items are rated on a scale of 0 (not at all confident) to 100 (highly confident) and averaged to obtain a total score. All study outcomes will be measured at baseline and at months 3, 6, 9 and 12 in both study groups (Fig. 1). To avoid anticipation of surveys potentially influencing responses, participants will only be informed that they will receive survey requests at random times over the study period.

### Analyses

Baseline data collection will include both demographic and MS-related information (Table 1); the same information will be collected at the end of the 12-month study period for all participants. All analyses will follow the intention-to-treat principle. The primary outcome will be analysed with repeated measures analysis of variance (ANOVA) and mixed effects models to compare GLTEQ scores between the intervention and control groups at 12 months. A mixed effects model will incorporate missing data under the assumption of missing at random. Although the GLTEQ is an ordinal scale, we will treat it as continuous because this is common practice for this measure [37] and will allow comparisons with the existing literature. Further within-subject effect comparison at 12 months will be done using ANOVA. The group-by-time interaction will be explored using a mixed effects model after controlling for potential covariates. Similar analyses will occur for the secondary outcomes.

**Table 1:** Baseline data collection variables

Variable	
Age (years)	
Sex	
Male	
Female	
Height	
Weight	
BMI	
Within normal limits	
Under normal limits	
Over normal limits	
Medical history	
Osteoarthritis	
Osteoporosis	
Coronary heart disease	
Stroke	
Diabetes	
Chronic respiratory disease	
Parkinson disease	
Cancer	
Other (list)	
Residence	
Urban	
Rural	
Type of MS	
Relapsing-remitting	
Secondary progressive	
Primary progressive	
Progressive relapsing	
Unknown	
Year of MS onset (e.g., first symptom)	
Year of MS diagnosis (by a neurologist)	
Most recent relapse (month/year)	
Walking aid or assistive device required?	
No	
Yes (if yes, list type)	
Employment	
Full-time (≥36 hours weekly)	
Part-time	
Casual	
Unemployed due to MS	
Retired	
Any falls in past 6 months?	
No	
Yes (frequency and severity)	
Disease-modifying therapy use	
Never	
Past (list)	
Current (list)	
Hospital Anxiety and Depression Scale (HADS) score	
Timed 25-ft walk (seconds)	
Nine-hole peg test (seconds)	
Symbol Digit Modality Test score	
Godin Leisure-Time Exercise Questionnaire score	
Multiple Sclerosis Impact Scale-29 v2 score	
Multiple Sclerosis Self-Efficacy Scale score	
Exercise Self-Efficacy Scale score	

*BMI* body mass index, *MS* multiple sclerosis

Using a conservative effect size of 0.3 for the primary outcome (change in GLTEQ), a power of 80%, and an alpha of 0.5, we estimate that 120 participants are needed for this study, allowing for 20% dropout. All statistical analyses will be conducted by the study biostatistician with use of SAS software (SAS Institute Inc., Cary, NC, USA), and all study data will be managed in REDCap (Vanderbilt, v6.7).

### Monitoring

This study, including the participant consent form, has received ethical approval from the University of Saskatchewan Biomedical Research Ethics Board. Because this is a low-risk intervention, no data monitoring review committee is required. However, the University of Saskatchewan Biomedical Research Ethics Board has the authority to audit the study at any time to ensure compliance with approved protocols. Monthly research meetings involving the research team will be held to discuss day-to-day management and organisation of the study, including participant recruitment, delivery of the intervention, and participant monitoring. Finally, a trial steering committee comprised of the principal investigators, co-investigators, patient and family advisors, funders, and other stakeholders will meet quarterly over the course of the study period to monitor the overall study conduct and progress.

### Dissemination

Study results will be shared with all relevant end users by a variety of methods. Results will be shared with study participants and the public through community presentations (live or webinars) and social media. These communications will be facilitated by various stakeholders, including the Multiple Sclerosis Society of Canada – Saskatchewan Division. Communication of study results will also be sent to policy and decision makers at the provincial health authority and government levels. Scientific publications and presentations will target researchers and healthcare professionals. A final de-identified dataset may be available from the researchers upon request.

## Discussion

In 2016, the number of MS cases globally was estimated to be over 2.2 million [38]. North America has the highest number of reported cases of MS, with a prevalence of 165 per 100,000 [38]. MS places a significant burden on both individuals and society due to its disabling, long-term nature; high healthcare use; and lost productivity [39, 40]. By 2031, MS-related healthcare expenses are projected to reach $2 billion annually in Canada [41]. Physical activity interventions consistently show an improvement in walking function based on clinical trial data [6] and should be further evaluated as cost-effective methods in the management of MS [41, 42].

Individuals with MS report that it is challenging for them to engage in physical activity often enough to gain health benefits and have indicated the need for support to initiate and maintain physical activity [5]. Recognising that increasing and maintaining physical activity levels in MS can be challenging, we consulted with individuals with MS and their families/caregivers during the development of the study design, intervention, outcome measures, and dissemination plan to ensure relevance and feasibility for participants. The result is a novel study design that combines behaviour change strategies with expert (neurophysiotherapy) support to increase physical activity levels in individuals with MS.

As with any study, there are potential limitations to consider. First, our primary outcome is a self-reported measure and may be susceptible to recall bias. The value of patient-reported outcomes is recognised by many organisations, including the US Food and Drug Administration, as they not only measure specific outcomes but also capture an individual’s perceptions of their health and experiences [43]. The use of patient-reported outcomes in MS research is also increasing [43, 44]. The GLTEQ is a validated measure that has been used extensively in MS research and only requires participants to recall the last 7 days of activity. We have also purposely not disclosed the timing of questionnaire distributions to participants to try to minimise any potential for the Hawthorne effect [45]. Because we are limiting our enrolment to ambulatory individuals with MS, the results will not be directly applicable to those who are non-ambulatory. However, we have attempted to increase the study generalisability with our very limited exclusion criteria and by allowing the study intervention to be delivered in locations and via methods that are most convenient to the participants.

Physical activity has been identified as a top research priority by the MS community [46–48]. Our study is designed to be both feasible and replicable in real-world settings and may lead to new knowledge that could significantly impact the management and overall health of individuals with MS.

## Trial status

Participant enrolment began on 19 July 2019. At the time of proof review (26 November 2019), enrolment is complete, and it is expected to be complete by December 2019.

## Supplementary information

**Additional file 1.** SPIRIT 2013 Checklist: Recommended items to address in a clinical trial protocol and related documents.

## Data Availability

The de-identified dataset analysed during the current study may be available from the researchers on reasonable request.

## Abbreviations

ANOVA: Analysis of variance
BCTT: Behavior Change Technique Taxonomy
BMI: Body mass index
GLTEQ: Godin Leisure-Time Exercise Questionnaire
IPAC-MS: Individualized Physiotherapy and Activity Coaching for Multiple Sclerosis
MS: Multiple sclerosis
SMSDP: Saskatchewan MS Drugs Program
